# High-Performance Asymmetric Optical Transmission Based on a Dielectric–Metal Metasurface

**DOI:** 10.3390/nano11092410

**Published:** 2021-09-16

**Authors:** Wenbing Liu, Lirong Huang, Jifei Ding, Chenkai Xie, Yi Luo, Wei Hong

**Affiliations:** Wuhan National Laboratory for Optoelectronics, Huazhong University of Science and Technology, 1037 Luoyu Rd, Wuhan 430074, China; D201780662@hust.edu.cn (W.L.); D201880718@hust.edu.cn (J.D.); M201972863@hust.edu.cn (C.X.); D202080923@hust.edu.cn (Y.L.)

**Keywords:** metasurface, asymmetric optical transmission, surface plasmon polaritons, Kerker conditions

## Abstract

Asymmetric optical transmission plays a key role in many optical systems. In this work, we propose and numerically demonstrate a dielectric–metal metasurface that can achieve high-performance asymmetric transmission for linearly polarized light in the near-infrared region. Most notably, it supports a forward transmittance peak (with a transmittance of 0.70) and a backward transmittance dip (with a transmittance of 0.07) at the same wavelength of 922 nm, which significantly enhances operation bandwidth and the contrast ratio between forward and backward transmittances. Mechanism analyses reveal that the forward transmittance peak is caused by the unidirectional excitation of surface plasmon polaritons and the first Kerker condition, whereas the backward transmittance dip is due to reflection from the metal film and a strong toroidal dipole response. Our work provides an alternative and simple way to obtain high-performance asymmetric transmission devices.

## 1. Introduction

Asymmetric optical transmission refers to different transmittance responses when a beam of light passes through a medium in forward and backward directions. Over the past few decades, due to its indispensable role in many optical systems, asymmetric transmission (AT) has attracted great attention in various applications, such as optical isolating [1,2], optical diodes [3,4], noise control or cancelation [5,6], systems for one-side detection/sensing [7,8], etc. One conventional way to achieve AT devices is to take advantage of non-reciprocity, which can be obtained by using magneto-optical materials [9,10] or nonlinear effects [11]. However, it is difficult to achieve on-chip integration due to their bulky size and the requirement of high threshold operating intensity.

Fortunately, the emergence of metamaterial provides an appealing alternative to control electromagnetic wave manipulations properties [12,13,14,15,16,17], and the discovery of the AT phenomenon based on metamaterial was first experimentally demonstrated in the microwave region by Fedotov et al. in 2006 [18]. Since then, various AT devices based on artificial structures have been proposed which use photonic crystals [19,20], subwavelength asymmetric gratings [21,22,23,24], chiral metamaterials [25,26,27] and metasurfaces [28,29,30], and the operation wavelengths have been covered from microwave to visible light [31,32,33]. These devices show promise to some degree; however, those using chiral metamaterials are usually complex and incorporate multilayer structures, whereas those using subwavelength asymmetric gratings are polarization sensitive. Therefore, it is still highly desirable to develop polarization-insensitive AT devices with simpler structures, polarization independence and higher contrast ratios between forward and backward transmittances. 

Here, we employ a dielectric–metal metasurface to realize high-performance asymmetric transmission (AT) for linearly polarized light at the near-infrared region. Its structural unit consists of a dielectric (Al_2_O_3_) disk and a thin layer of gold (Au) film on a SiO_2_ substrate. Simulation results show that it supports a forward transmittance peak (with a transmittance of 0.70) and a backward transmittance dip (with a transmittance of 0.07) at 922 nm. The occurrence of a forward transmission peak and backward transmission dip at the same wavelength notably enhances operation bandwidth and the contrast ratio between the forward and backward transmittances. This is quite different from previously reported works: to the knowledge of the authors, such a phenomenon has not been reported yet. Here, it is revealed that unidirectional surface plasmon polaritons (SPPs) excitation and Kerker conditions are responsible for the emergence of the forward transmittance peak, while the reflection from the metal layer and strong toroidal dipole response are responsible for the backward transmittance dip. 

The rest of the paper is organized as follows: In Section 2, we present the device structure and operation principle. In Section 3, we first interpret the origin of the AT effect by using unidirectional excitation and tunneling of SPPs and Kerker conditions. Finally, a conclusion is given in Section 4.

## 2. Materials and Methods

**Figure 1:**
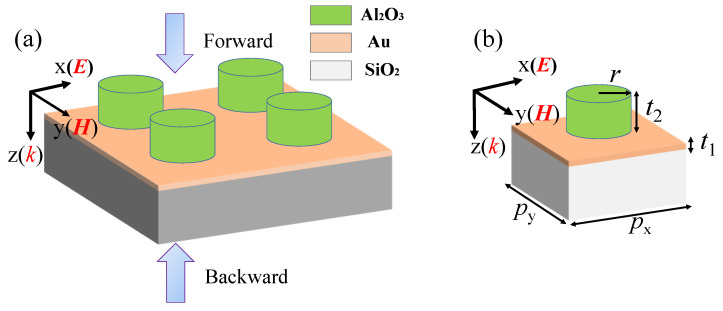
Schematic diagrams of the proposed dielectric–metal metasurface. (**a**) Perspective view of the structural unit arrays of the dielectric–metal metasurface. (**b**) The optimized geometrical parameters of one unit: *p_x_* = *p_y_* = 700 nm, *t*_1_ = 20 nm, *t*_2_ = 200 nm, *r* = 200 nm.

Figure 1a schematically shows the structural unit array of the proposed dielectric–metal metasurface, which consists of Al_2_O_3_ dielectric disks and a thin layer of gold (Au) film on a SiO_2_ substrate. Figure 1b is for one structural unit. The radius of Al_2_O_3_ disk is *r* = 200 nm and the thicknesses of the Au layer and Al_2_O_3_ disk are *t*_1_ = 20 nm and *t*_2_ = 200 nm, respectively; structural units are periodically arranged with period *p_x_* = *p_y_* = 700 nm. Compared with previous AT devices based on metal–insulator–metal (M–I–M) [22] or metal–metal–metal (M–M–M) subwavelength grating [23], our design can also be viewed as a kind of meta-grating with a relatively simple and easy-to-fabricate structure that can be easily integrated with other optoelectronic devices. 

In addition, it can be observed that the proposed device has rotational symmetry, meaning it can work under both x- and y-linearly polarized light as well as circularly polarized light. Next, for simplicity, we will just discuss the case of x-polarized incident light.

The operation mechanism of the AT device mainly involves unidirectional SPPs excitation and Kerker conditions. Therefore, we introduce these two aspects below.

(1) Unidirectional excitation of SPPs

To begin, we explain how unidirectional SPP excitation is produced. When x-polarized incident light illuminates the device in the forward direction (i.e., along the positive z-axis), the light first impinges on the periodically arranged dielectric disks which provide the required wave vector for efficient excitation of surface plasmon polaritons (SPPs), and thus, help electromagnetic energy tunnel through the Au film. By contrast, in the case of backward illumination, the light first hits the Au film. Lacking a periodic structure to provide an extra wave vector to excite SPPs, the device obtains low backward transmittance, and most of the electromagnetic energy is reflected. 

(2) Kerker conditions

Kerker conditions were first proposed by M. Kerker et al. [34] to study electromagnetic scattering by magnetic spheres with zero backward or zero forward scattering arising from the interference between electric and magnetic dipole modes. A direct conclusion is that when electric dipole (ED) and magnetic dipole (MD) resonances oscillate in-phase with equal magnitudes, the scattered fields are mainly in the direction of the incoming wave with zero-backward radiated power, which is named as the first Kerker condition. Correspondingly, when the two dipoles are of equal magnitude but oscillate out of phase, they may lead to zero forward radiation, which is named as the second Kerker condition. To date, this theory has been expanded to study directional scattering from non-spherical nanostructures containing electric and magnetic multipole resonant modes [35,36].

On the other hand, as is well known, a dielectric nanostructure, such as a nanosphere or a nanodisk, can simultaneously support electric and magnetic responses [37,38,39]. Therefore, the Al_2_O_3_ dielectric disk of a suitable thickness also inherently possesses a strong electric and magnetic dipole or multipolar moments, and the overlap between the ED and MD resonances can greatly enhance forward transmittance at the operation wavelength if we carefully design the metasurface by deliberately employing Kerker conditions. Next, we discuss how Kerker conditions work in our AT device.

When an x-polarized wave normally illuminates the metasurface along the positive z-axis (i.e., the forward propagation direction), the light first hits the dielectric disk array and excites an electric dipole ***p*_x_** along the x-axis within each disk. Different from a plasmonic structure, which needs a special profile to excite a circular current to support an equivalent magnetic dipole response, the dielectric disk itself can simultaneously support electric dipole and magnetic dipole responses. Therefore, a magnetic dipole moment ***m*_y_** along the y-axis is induced inside the Al_2_O_3_ dielectric disk. The normalized forward/backward (i.e., along the positive or negative z-axis) scattering cross-section of the structure unit can be expressed as [40]:

(1) $$Q=\frac{k^{4}}{4\pi \varepsilon^{2}A{\left|E_{\mathrm{inc}}\right|}^{2}}{\left|p_{x}\pm \frac{\sqrt{\varepsilon_{r}}m_{y}}{c}\right|}^{2},$$

where *k* is the wavenumber in a background material with electric permittivity *ε* = *ε*_0_*ε*_r_, *c* is the speed of light in free space, and |***E***_inc_| is the electric-field amplitude of the incident wave. *A* is the geometrical cross-section, and the symbols ‘±’ represent forward and backward scattering cross sections, respectively.

According to Equation (1), zero backward scattering occurs at some wavelengths when the first Kerker condition is met, which is expressed as:

(2) $$p_{x}-\frac{\sqrt{\varepsilon_{r}}m_{y}}{c}=0,$$

In this case, the scattered field is mainly in the direction of the incoming wave, leading to a zero reflection, and a high transmittance is achieved if optical absorption is also low. 

Similarly, zero forward scattering takes place at some wavelengths when the second Kerker condition is fulfilled, which is written as:

(3) $$p_{x}+\frac{\sqrt{\varepsilon_{r}}m_{y}}{c}=0,$$

In this case, the scattered field mainly propagates opposite to the direction of the incoming wave, leading to a zero transmission.

At other wavelengths, if neither the first Kerker condition nor the second Kerker condition is met, then the light will be partly transmitted and partly reflected.

## 3. Results and Discussion

To verify the AT behavior of the proposed dielectric–metal metasurface, we carry out full three-dimensional (3D) finite-difference time-domain (FDTD) simulations by employing the FDTD solver from Lumerical, Inc. to simulate its electromagnetic response and scattering characteristics. Periodic boundary conditions are applied in the x- and y-directions, and a perfectly matched layer condition is applied in the z-direction. The substrate is silica (SiO_2_), the dielectric disk is Al_2_O_3_, and their permittivity values are inferred from Palik refractive index database values [21]. The thin metal layer is gold, and its dielectric constants are taken from Johnson and Christy data, which can also be obtained in the material database from the simulation software. Mesh refinement is used for both the thin metallic film and Al_2_O_3_; the mesh sizes in the *z*-direction are set as *d*z = 2 nm and *d*z = 10 nm, respectively, while the mesh size in the *x*- and *y*-directions are both set as *d*x = *d*y = 10 nm.

### 3.1. High-Performance Asymmetric Transmission

**Figure 2:**
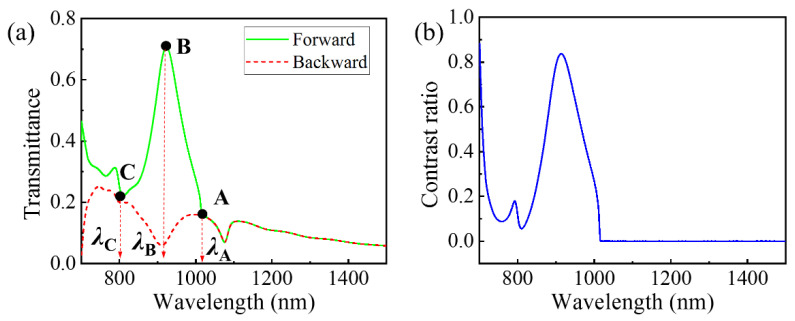
(**a**) Forward and backward transmittance spectra of the metasurface. (**b**) Contrast ratio versus wavelength. The marked wavelengths are *λ*_A_ = 1017 nm at point A, *λ*_B_ = 922 nm at point B, and *λ*_C_ = 800 nm at point C.

The simulated transmittance spectra under forward (green solid line) and backward (red dashed line) illumination are shown in Figure 2a. It can be clearly seen that the asymmetric transmission region exists at a cut-off wavelength (i.e., *λ*_A_) corresponding to the Wood–Rayleigh anomaly wavelength given by *λ* = *np* [41], where *n* is the refractive index of the substrate and *p* is the structural period. In our case, *n* and *p* are 1.45 and 700 nm, respectively. Because *λ* = *np* = 1015 nm, it coincides well with the simulation result *λ*_A_ = 1017 nm. It should be noted that the cut-off wavelength divides the wavelength domain into a diffraction region (*λ* < *λ*_A_) and a non-diffraction region (*λ* > *λ*_A_). Furthermore, the asymmetric transmission phenomenon occurs in the diffraction region [41,42].

Moreover, a very interesting phenomenon can be clearly seen in Figure 2a, where the forward transmission peak and backward transmission dip occur at the same wavelength *λ*_B_ = 922 nm. When propagating in the forward direction, the optical wave at this wavelength is a transmittance peak, with a transmittance of 0.70. However, when this wave backwardly illuminates the metasurface, a transmittance dip is observed, with a very low transmittance of 0.07. This is quite different from previously reported AT works. To the best of our knowledge, such a phenomenon has not been reported yet.

A good asymmetric transmission device requires not only a higher forward transmittance but also a larger contrast ratio and wide operation bandwidth. A contrast ratio is used to evaluate the degree of asymmetric transmission, which is defined as:

(4) $$\mathrm{contrast}\;\mathrm{ratio}=\frac{\left|T_{f}-T_{b}\right|}{T_{f}+T_{b}},$$

where *T*_f_ and *T*_b_ are the forward and backward transmittances, respectively. 

As shown in Figure 2b, the contrast ratio reaches 0.82 at *λ*_B_ = 922 nm, and the full width at half maximum (FWHM) is 100 nm, which means the proposed dielectric–metal metasurface has a large wide operation bandwidth. The reason for the high contrast ratio and big bandwidth mainly lies in the fact that the forward transmission peak and backward transmission dip occur at the same wavelength.

In what follows, we will further reveal how the device achieves high-performance asymmetric transmission. 

### 3.2. Mechanism Analyses on Asymmetric Transmission

**Figure 3:**
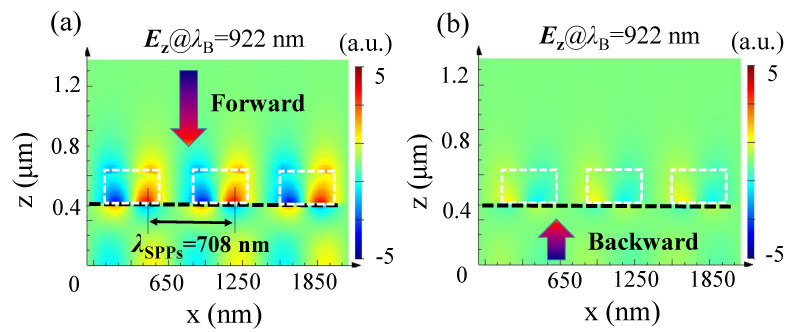
Electric-field ***E*_z_** distributions along the x-z plane at *λ*_B_ = 922 nm under forward (**a**) and backward (**b**) illumination.

To demonstrate how the unidirectional excitation of SPPs works in our device, Figure 3 shows the corresponding ***E*_z_** electric-field distributions at *λ*_B_ = 922 nm, where the white dotted lines mark the locations of the dielectric disks and the black dotted line marks that of the metal layer. As can be clearly seen in Figure 3a, under forward illumination, SPPs with wavelength *λ*_SPP_ = 708 nm are excited in the dielectric–metal interface, then tunnel through the Au film and, finally, enter the SiO_2_ substrate, meaning a high forward transmittance is obtained. In contrast, for backward incident light at *λ*_B_ = 922 nm, the electric field intensity is too weak to see obvious excitation of SPPs, as shown in Figure 3b. This result occurs when the incident direction is reversed because the light first hits the Au film. On the one hand, there are no periodically arranged resonators to provide the extra wave vector to efficiently excite SPPs. On the other hand, the Au film in this case works as a high-efficiency reflector. As a result, most of the electromagnetic energy is reflected by the Au film. 

However, the appearance of SPPs does not guarantee that the corresponding transmittance will be very high. Next, we will further investigate how the device can achieve a forward transmission peak and backward transmission dip at the same wavelength of 922 nm.

#### 3.2.1. Role of Kerker Conditions in the Realization of the Forward Transmission Peak 

Above, we have offered a preliminary analysis on the origin of the asymmetric transmission; now, we further investigate how the forward transmission peak is achieved at *λ*_B_ = 922 nm. As mentioned earlier, Kerker conditions are usually adopted to explain electromagnetic scattering characteristics in many metasurface systems, such as perfect transmission [36,43], and asymmetric transmission (AT) [21,22].

**Figure 4:**
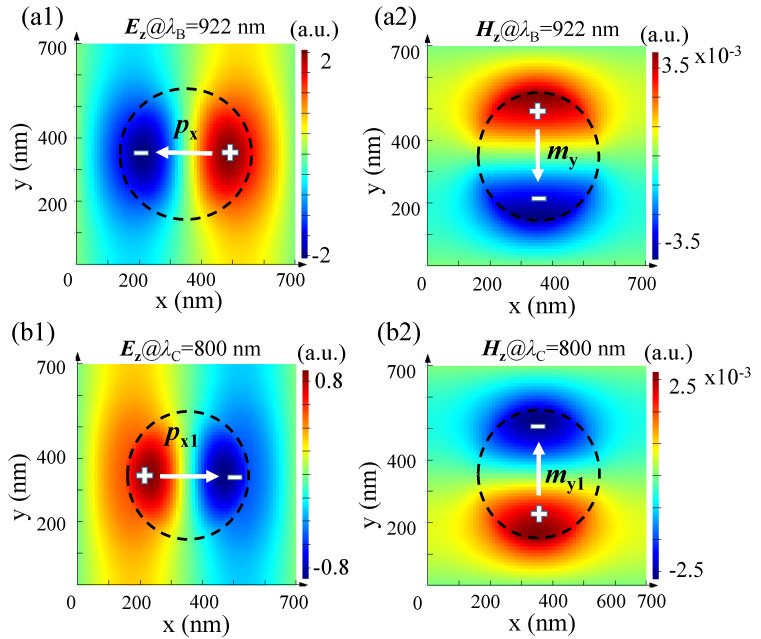
Electromagnetic field distribution in the x–y plane for forward incidence light. (**a1**) Electric dipole and (**a2**) magnetic dipole at *λ*_B_ = 922 nm. (**b1**) Electric dipole and (**b2**) magnetic dipole at *λ*_C_ = 800 nm. The black dotted line marks the outline of the Al_2_O_3_ disk. The white arrow stands for the direction of the electric or magnetic dipole.

Here, in order to investigate how Kerker conditions are satisfied in our AT device, we choose two specific wavelengths for comparative analysis: one is *λ*_B_ = 922 nm, at which forward transmittance has a maximal value of 0.70, and the other is *λ*_C_ = 800 nm, at which forward transmittance reaches a minimal value of 0.21. Figure 4(a1,a2) and Figure 4(b1,b2) plot their electromagnetic field distribution profiles in the x–y plane under forward incidence.

At *λ*_B_ = 922 nm, when the metasurface is forwardly illuminated by x-polarized light, a strong ***E*_z_** field is excited in the dielectric disk (marked by the black dashed line in Figure 4(a1)), and the stimulated positive (marked as ‘+’) and negative (marked as ‘−’) charges are formed, which give rise to an electric-dipole moment ***p*_x_** oscillating along the negative x-axis (the white arrow stands for the direction of the electric dipole). Meanwhile, as shown in Figure 4(a2), the dielectric disk also supports a magnetic dipolar ***m*_y_** along the negative y-axis. ***p*_x_** and ***m*_y_** are both along the negative directions, signifying that the radiated fields from ***p*_x_** and ***m*_y_** oscillations are nearly in phase; hence, their overlap causes strong suppression in reflection, which implies that the first Kerker condition is satisfied and zero backward scattering occurs. As a consequence, a forward transmittance peak, with a transmittance of 0.70, appears at *λ*_B_ = 922 nm.

At *λ*_C_ = 800 nm, Figure 4(b1) shows electric dipole ***p*_x1_** is along the positive direction of the x-axis, and the induced magnetic-dipole moment ***m*_y1_** is along the positive y-axis, as shown in Figure 4(b2). Therefore, the radiated fields from ***p*_x1_** and ***m*_y1_** oscillations are also nearly in phase. However, by comparing the electric- and magnetic-dipole distributions at *λ*_C_ (see Figure 4(b1,b2)) with those at *λ*_B_ (see Figure 4(a1,a2)), one can see that the amplitude of ***p*_x1_** at *λ*_C_ = 800 nm is much lower than that of ***p*_x_** at *λ*_B_ = 922 nm, and the amplitude of ***m*_y1_** at *λ*_C_ = 800 nm is close to that of ***m*_y_** at *λ*_B_ = 922 nm. Nevertheless, the large difference in amplitudes of ***p*_x1_** and ***m*_y1_** infers that the interaction between them cannot lead to a good interference to obtain high forward transmittance at *λ*_C_ = 800 nm. As a consequence, the forward transmittance at *λ*_C_ = 800 nm has a value of only 0.21.

#### 3.2.2. Realization of the Low Backward Transmittance Dip

After analyzing the origin of the forward transmittance peak at *λ*_B_ = 922 nm, we now continue to investigate the origin of the low backward transmittance dip at the same wavelength.

In the discussion of Figure 3b, we already explained that the lack of efficient excitation of SPPs and the back reflection by the Au film contribute to the low backward transmittance. However, this cannot explain why a transmittance dip with a near-zero transmittance appears at *λ*_B_. 

We know that geometrical parameters of a device determine its electromagnetic (EM) response, thus affecting its scattering characteristics when it interacts with electromagnetic waves [44,45]. As a kind of scattering, the characteristic transmittance curve is also directly affected by different electromagnetic responses. In this regard, it is common to study their resonant properties in terms of electric and magnetic multipoles, which can be derived from the Taylor expansion of their EM fields and potentials [46]. Here, we only consider electric dipole (ED), magnetic dipole (MD), electric quadrupole (EQ), magnetic quadrupole (MQ) and toroidal dipole (TD), because higher-order electric and magnetic resonance modes cannot be excited. In a Cartesian coordinate system, the multipole decomposition can be, respectively, written as [47]:

(5) $$P=\frac{1}{i\omega }\int jd^{3}r,$$

(6) $$M=\frac{1}{2c}\int \left(r\times j\right)d^{3}r,$$

(7) $$QE_{\alpha \beta }=\frac{1}{i\omega }\int \left[\left(r_{\alpha }j_{\beta }+r_{\beta }j_{\alpha }\right)-\frac{2}{3}\left(r\cdot j\right)\right]d^{3}r,$$

(8) $$QM_{\alpha \beta }=\frac{1}{3c}\int \left[{\left(r\times j\right)}_{\alpha }j_{\beta }+{\left(r\times j\right)}_{\beta }j_{\alpha }\right]d^{3}r,$$

(9) $$T=\frac{1}{10c}\int \left[\left(r\cdot j\right)r-2r^{2}j\right]d^{3}r.$$

where *c* is the speed of light, ***j*** is current density, *ω* is the angular frequency of the electromagnetic wave and ***r*** is the position vector from the origin to point (x, y, z) in a Cartesian coordinate system (*α*, *β* = x, y, z). ***P***, ***M***, ***QE***_*αβ*_, ***QM***_*αβ*_, and ***T*** are the electric dipole (ED) moment, magnetic dipole (MD) moment, electric quadrupole (EQ) moment, magnetic quadrupole (MQ) moment and toroidal dipole (TD) moment, respectively.

The corresponding radiated light powers are calculated by [47]:

(10) $$I_{P}=\frac{2\omega^{4}}{3c^{3}}{\left|P\right|}^{2},$$

(11) $$I_{M}=\frac{2\omega^{4}}{3c^{3}}{\left|M\right|}^{2},$$

(12) $$I_{T}=\frac{2\omega^{6}}{3c^{5}}{\left|T\right|}^{2},$$

(13) $$I_{QE}=\frac{\omega^{6}}{5c^{5}}{\sum \left|{\mathrm{QE}}_{\alpha \beta }\right|}^{2},$$

(14) $$I_{QM}=\frac{\omega^{6}}{20c^{5}}{\sum \left|{\mathrm{QM}}_{\alpha \beta }\right|}^{2}.$$

**Figure 5:**
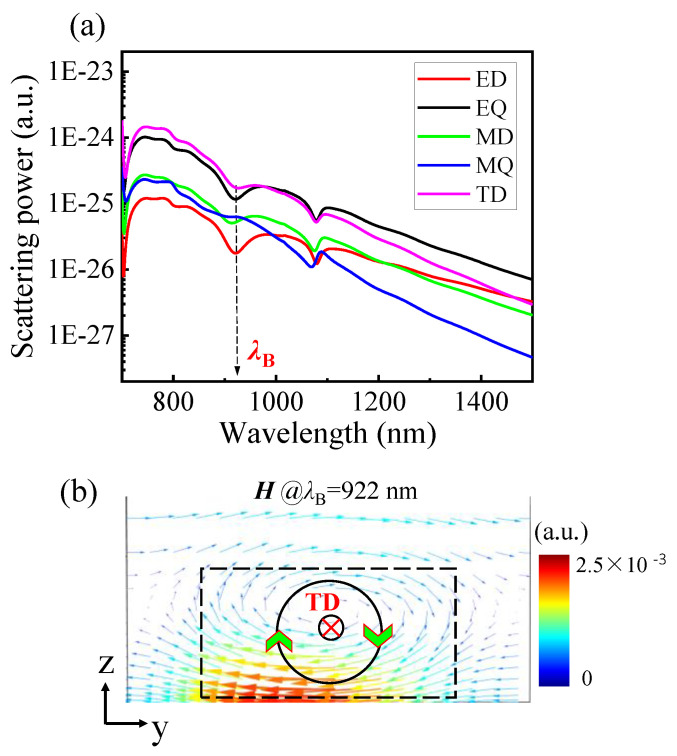
(**a**) Scattering spectra for ED, MD, EQ, MQ, and TD modes under backward illumination. (**b**) Cross-sectional vectorial (y–z plane) for the magnetic-field (***H***-field) maps for the excitation of toroidal dipole (TD). The black dashed line marks the outline of Al_2_O_3_ disk, while the black circular lines along the circumference indicate toroidal moment. The green arrow marks the circular magnetic field direction, and the direction of TD is shown by the symbol 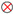.

According to Equations (10)–(14), we calculate electromagnetic multipolar dipoles under backward illumination and present the results in Figure 5a. As shown, near *λ*_B_ = 922 nm, the toroidal dipole (TD) has the strongest scattering power while the electric quadrupole (EQ) ranks second; other electromagnetic multipoles account for a small proportion, especially the electric dipole (ED) and the magnetic dipole (MD), which are strongly suppressed. These results demonstrate that the resonance at *λ*_B_ = 922 nm is dominated by the toroidal dipole moment.

As we know, the toroidal dipole (TD), as an independent family of elementary electromagnetic source, corresponds to electric currents flowing on the surface of a torus. However, toroidal dipole response in electrodynamics is often masked by the more dominant electric and magnetic multipoles at a similar frequency. Recently, due to its nonradiating feature, toroidal dipole has attracted increasing attention in metasurface research, and it provides many interesting phenomena with enhanced light–matter interactions and applications in spasers [48], ultrasensitive biosensing [49] and nonlinear effects [50]. However, the application of toroidal dipole in asymmetric transmission has not been reported yet. As can be clearly seen in Figure 5a, the scattering of the toroidal dipole (TD) corresponds to a dip at *λ*_B_, and other electromagnetic poles, except for the magnetic quadrupole (MQ), also exhibit a resonance dip at this wavelength. These dips demonstrate the appearance of a low backward transmittance dip at *λ*_B_ = 922 nm.

Based on the above discussion, we believe that the lack of efficient excitation of SPPs, the back reflection by the Au film and the dominant toroidal dipole response together contribute to the appearance of a backward transmittance dip at *λ*_B_ = 922 nm.

To further verify the existence of TD, the cross-sectional (y–z plane) magnetic-field (***H***-field) at *λ*_B_ = 922 nm is plotted in Figure 5b, in which the black dashed line shows the position of the Al_2_O_3_ dielectric disk. As shown, the poloidal magnetic field spins in a clockwise path in the Al_2_O_3_ dielectric disk, which can explain the existence of the toroidal dipole (TD), represented by the black circle line in Figure 5b, while the green arrow marks the circular magnetic field direction.

Furthermore, from Figure 5b, one can see that the circulating magnetic fields are mainly located inside the dielectric disk; hence, changing the thickness of the dielectric disk will have a significant impact on the transmittance response.

#### 3.2.3. Effect of the Thickness of the Dielectric Disk and Au Layer on Asymmetric Transmission

Besides the disk thickness mentioned above, which has a significant effect on transmission spectrum, the metal layer’s thickness is also crucial because it plays a key role in the tunneling of SPPs and impacts transmittance spectra. Therefore, it is necessary to choose suitable thicknesses for the Au film and dielectric disk.

**Figure 6:**
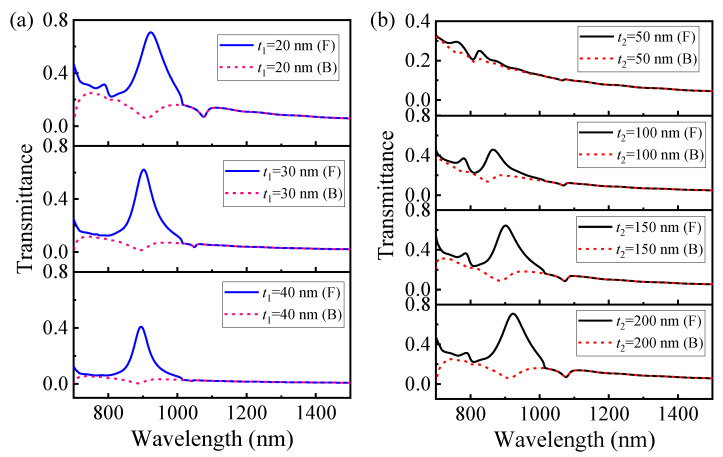
Forward (F) and backward (B) transmittance spectra for different geometric parameters. (**a**) Au layer thickness *t*_1_; (**b**) dielectric disk thickness *t*_2_.

Firstly, as shown in Figure 6a, when the thickness of the gold (Au) layer, *t*_1_, increases from 20 nm to 40 nm by a step of 10 nm (while other geometric parameters remain unchanged), both the forward (F) and backward (B) transmittance spectra tend to decrease. Obviously, in the case of forward incidence, it is more difficult for the SPPs to tunnel through a thicker Au layer, whereas in the case of backward incidence, a thicker Au layer reflects more light. As a result, both forward and backward transmission decreases with the increase in Au thickness.

Secondly, as shown in Figure 6b, when the thickness of the dielectric disk *t*_2_ is 50 nm, the maximal forward transmittance is only 0.3. With the increase in *t*_2_ (while other geometric parameters remain unchanged), the peak value of forward transmittance increases gradually. Most notably, when *t*_2_ increases to 200 nm, the maximal forward transmittance is close to 0.70 at 922 nm. This can be understood as follows: when *t*_2_ is small, it is hard to excite the annular displacement current inside the dielectric disk; thus, an effective magnetic response is unable to form [51,52], which affects the interference effect between electromagnetic multipolar moments. Therefore, in this paper, we choose *t*_2_ = 200 nm as the optimization thickness for the dielectric disks.

In addition, under backward illumination, when *t*_2_ increases from 50 nm to 200 nm, the transmission dip gradually redshifts, and its corresponding transmittance also decreases. This is especially noticeable when *t*_2_ increases to 200 nm, for its value is only 0.07 at *λ*_B_ = 922 nm. This is because the toroidal dipole response gradually rises to the dominant position; thus, it further suppresses transmission and, finally, leads to a low transmittance dip at *λ*_B_ = 922 nm.

## 4. Conclusions

In summary, we propose and numerically demonstrate a dielectric–metal metasurface that can work as a high-performance asymmetric transmission (AT) device in the near-infrared region. The simulation results show that it can support a forward transmittance peak (with a transmittance of 0.70) and a backward transmittance dip (with a transmittance of 0.07) at wavelength 922 nm. The occurrence of a forward transmission peak and backward transmission dip at the same wavelength notably enhances the operation bandwidth and the contrast ratio between the forward and backward transmittances, and this feature is quite different from previously reported devices. The physical mechanism behind this extraordinary phenomenon is investigated with respect to the unidirectional excitation of surface plasmon polaritons (SPPs), Kerker conditions and the electromagnetic multipole decomposition method. To be specific, when the light is forwardly incident on the proposed dielectric–metal metasurface, the dielectric disk and the metal film excite SPPs; meanwhile, the electric and magnetic responses within the dielectric disk satisfy the first Kerker condition so that the electromagnetic energy can tunnel through the metal layer with the assistance of SPPs, realizing a transmittance peak at 922 nm. However, when the incident direction is reversed, the light cannot pass the device due to the lack of effective SPPs excitation, resulting in most electromagnetic energy being reflected by the metal layer. At the same time, the nonradiating toroidal dipole dominates in the far-field scattering powers, and it further promotes the formation of a transmittance dip at 922 nm.

In view of its simple structure and excellent performance, we believe that the proposed metasurface shows extensive versatility for potential applications including, but not limited to, noise control, one-side detection, optical beam splitters and other photonic devices.

## Data Availability

Data are contained within the article.
